# Improving cardiovascular health by replacing salt with low-sodium salt substitutes: a synthesis of existing evidence

**DOI:** 10.12968/bjca.2023.0038

**Published:** 2023-07-19

**Authors:** Areej Mohamed, Zundus Ali-heybe, Oliver Hamer, James Hill

**Affiliations:** 1 Blackpool Teaching Hospitals NHS Foundation Trust, Blackpool, UK; 2 University of Central Lancashire, Preston, UK; 3 National Institute for Health and Care Research Applied Research Collaboration Northwest Coast, UK

**Keywords:** Systematic review, Hypertension, Cardiovascular, Blood pressure, Salt, Nursing

## Abstract

Globally, hypertension is a significant contributor to cardiovascular, renal diseases and death. Increased dietary salt intake raises the risk of hypertension, which in turn raises the risk of cardiovascular disease, stroke, and kidney disorders. A practical approach to minimising salt intake to lower blood pressure is to use low salt sodium substitutes. However, there is some evidence that salt substitutes may lead to adverse events such as hyperkalaemia, which can cause arrythmias and cardiac arrests. Existing evidence is unclear as to whom is at risk of harm from low sodium salt substitutes, and thus there is now a need for a concise synthesis of evidence to guide healthcare practitioners. The aim of this commentary is to summarise the efficiency of substituting low sodium salt substitutes with regular salt for lowering blood pressure in adult, children, and pregnant women.

## Introduction

Globally, hypertension is a significant contributor to increase risk of renal diseases and death (Kjeldsen 2018). Furthermore, in adult populations, hypertension raises the risks of heart failure, coronary artery disease, and stroke (Kjeldsen 2018). The current standard threshold for suspecting hypertension is a clinical systolic blood pressure sustained above or equal to 140 mmHg, or diastolic blood pressure sustained above or equal to 90 mmHg (or both) (Jones et al. 2020). In the United Kingdom, the prevalence of adults with high blood pressure is approximately 26% for women and 31% for men, affecting more than one in four adults (approximately 12.5 million of the population) (NICE 2023). Hypertension is thought to be caused by various factors, including age, inadequate dietary potassium intake, excessive sodium consumption, obesity, alcohol consumption, inactivity, genetic predispositions, and adverse intrauterine environments (e.g., gestational hypertension or pre-eclampsia) (Oparil et al. 2018; Te Riet et al. 2015). One factor which has been found to have a direct relationship with blood pressure values is that of excessive sodium consumption (Mente et al. 2014).

Increased dietary salt intake raises the risk of hypertension, which in turn raises the risk of cardiovascular disease, stroke, and kidney disorders (Grillo et al. 2019; Mente et al. 2014). The World Health Organisation recommends intake of less than five grams salt per day to reduce early mortality and morbidity (WHO 2012). A practical approach to minimising salt intake for the purpose of lowering blood pressure (and avoid the adverse effects of high blood pressure), is to use low salt sodium substitutes (LSSS) (Neal et al. 2021). Salt substitutes have been suggested to be effective at improving clinical outcomes including reducing rates of stroke, major cardiovascular events, and death (from any cause) (Neal et al. 2021). However, there is some evidence that salt substitutes may lead to adverse events such as hyperkalaemia (high potassium), which can increase the risk of patient harm in the form of arrythmias and cardiac events (Adrian et al. 2019). That said, existing evidence lacks clear guidance for healthcare professionals and thus there is a need for a concise syntheses for practitioners (Brand et al. 2022).

### Aim of commentary

This commentary aims to critically appraise the methods used within the Cochrane review by Brand et al (2022) on effectiveness of low-sodium salt substitutes and expand upon the findings in the context of clinical practice.

## Methods used by Brand et al, 2022

A comprehensive search strategy including seven databases was conducted (i.e., MEDLINE, CINAHL, Cochrane register, Web of science, Embase, ClinicalTrials.gov and the WHO International Clinical Trials Registry Platform) (Brand et al. 2022). The literature search was conducted from conception to August 2021 with no language or publication restrictions. Prospective analytical cohort studies, cluster random controlled trials and randomised controlled trials (RCTs) that compared any type and technique of LSSS implementation with the usage of ordinary salt were considered for inclusion. Two independent reviewers carried out abstract, title, full paper screening, and data extraction. Additionally, an assessment of bias was conducted by two reviewers independently using the Cochrane RoB tool (Higgins et al. 2011). Any disagreements were resolved by discussion with a third reviewer. An assessment to establish the certainty of evidence for each outcome (rating of certainty) was conducted using the Grading of Recommendations Assessment, Development and Evaluation (GRADE) (Guyatt et al. 2011). Cross-over RCTs and cluster RCTs with fewer that two intervention and two control clusters were excluded (Brand et al. 2022). Additionally, studies with multi-component interventions were also excluded if the additional intervention components were not aimed primarily at promoting LSSS use (Brand et al. 2022). Meta-analysis was undertaken using a random-effects model calculating risk ratios and mean difference when combining data across multiple studies (Brand et al. 2022).

## Results

### Outcomes in adult population (18 years and older)

Moderate certainty evidence indicated that adults who consumed LSSS had a significant fewer non-fatal stroke, non-fatal acute coronary syndrome, reduced cardiovascular mortality, lower systolic blood pressure and diastolic blood pressure when compared to those who consumed regular salt. Non-graded evidence indicated that adults who consumed LSSS had a significant reduction in risk of all-cause mortality, antihypertensive medication use and microalbuminuria. Furthermore, adults receiving LSSS interventions had improved blood pressure control (GRADE: Very low) and 24-h urinary potassium excretion (non-graded) compared to those receiving regular salt. However, moderate quality evidence indicated that adults receiving LSSS interventions had slightly increased blood potassium compared to those receiving regular salt (See table 1 for full results) (Brand et al. 2022).

There was no evidence of difference in instances of stroke mortality (GRADE: Very low), hyperkalaemia, hypokalaemia (GRADE: Moderate), hypertension (GRADE: Low), macroalbuminuria (non-graded), blood triglycerides (non-graded), total blood cholesterol (non-graded) or cardiovascular events (GRADE: Very low) in adults who consumed LSSS compared to those receiving regular salt (Brand et al. 2022).

Notably, all included studies excluded participants whereby it was known that an increased intake of potassium could cause harm (e.g., patients with chronic kidney disease) (Brand et al. 2022).

### Outcomes in children (2 to < 18 years)

There was very low-quality evidence that children who received bread containing LSSS compared to those receiving bread containing regular salt had a slightly higher BMI at four months (non-graded). There was no evidence of difference in diastolic or systolic blood pressure (GRADE: Very low), 24-h urinary sodium secretion (non-graded) or potassium secretion (non-graded) within children who received bread containing LSSS compared to those receiving bread containing regular salt (see table 2 for full results) (Brand et al. 2022).

### Outcomes in pregnant women

No eligible studies were found to provide a comparison of low-sodium salt substitutes with regular salt or no intervention in pregnant women (Brand et al. 2022).

### Commentary

Using the AMSTAR 2 critical appraisal tool for systematic reviews, all 16 criteria were found to be satisfactory for this review (seen in Table 3) (Shea et al. 2017). Based on this appraisal, it is deemed that the systematic review provides a comprehensive synthesis of the relevant studies in relation to the research question.

### Implications for practise

In the adult population, the small mean differences in diastolic and systolic blood pressure favoured LSSS when compared to a regular salt diet (Brand et al. 2022). While these differences were statistically significant, they were not deemed clinically significant for reducing key clinical outcomes (e.g., cardiovascular events, based on moderate-certainty evidence). This was because the range of pooled mean differences in blood pressure (as indicated by the 95% confidence intervals) were not judged to be clinically beneficial based on previously established cut offs (e.g., reduction of 5 mmHg DBP and reduction of 10 mmHg SBP) (Ettehad et al. 2016; Thomopoulos et al. 2017). That being said, smaller mean reductions achieved in the population-level interventions may have a greater positive impact than larger reductions in fewer at-risk patients (Verbeek et al. 2021). For healthcare professionals, the observed reduction in blood pressure from low-sodium salt supplementation is likely to correspond to the prevention of approximately 35 to 83 stroke deaths per 100,000 patients, per year (aged 50 years and over) (Brand et al. 2022). LSSS is likely to slightly decrease non-fatal ACS occurrences, incidence of CVS mortality, and reduce non-fatal strokes in patients with high blood pressure (Brand et al. 2022). Although reductions were considered small and may not be clinically important at individual level, they are deemed important from a population perspective (Brand et al. 2022). These findings are consistent with other systematic reviews that have analysed the effects of low sodium salt supplementation in adult populations (Adrian et al. 2019; Jin et al. 2020).

There are several limitations that need to be considered before any changes to practise may be implemented. Firstly, the generalisability of the findings are limited as the majority of the participants in the studies had high blood pressure or were known hypertensives (Brand et al. 2022). In addition, all studies included in the review excluded high risk participants with CKD, type 1 or 2 diabetes mellitus, impaired renal function or those using potassium sparing medications. Literature has established that patients with these conditions are at risk of harm with increased potassium intake, and so the effect and safety of LSSS to these patients are unknown (Brand et al. 2022). Notably, the exclusion of these patients limits the generalisability of the findings, specifically as it relates to these population subgroups.

A further limitation that needs to be considered is that the risk of harm associated with LSSS supplementation (and the subsequent increase in potassium intake) is largely unclear because few studies reported adverse events (those that did were judged to be high risk of bias). That said, the review found no evidence of difference in adverse events of hyperkalaemia. This finding must be interpretated with caution because only a small number of studies reported the outcome, and those that did utilised varying standards to classify hyperkalaemia (Brand et al. 2022). Due to these limitations, a population-level implementation should be conducted with caution, excluding patients whereby increased intake of potassium could cause harm, and should be guided by further high-quality evidence (Brand et al. 2022).

At present, the findings suggests that there is no evidence of difference in children’s blood pressure from LSSS compared to ordinary salt. Consequently, there is currently no evidence to support changes in clinical practise or dietary guidance to utilise LSSS for reducing blood pressure in children. Similarly, there is a dearth of evidence to support clinical practise changes for the implementation of LSSS in place of ordinary salt for pregnant women (Brand et al. 2022).

### Implications for future research

The current findings identified several gaps in the evidence that require further research (Brand et al. 2022). Firstly, the majority of adult participants in this study were either known hypertensives or had high blood pressure at the time of implementation (Brand et al. 2022). To better understand the effectiveness and safety of LSSS in people with normal blood pressure, further studies are required that include a sample who are representative of the general population (with blood pressure in the healthy range). Secondly, to improve the quality of new research in this area, trial protocols should be registered detailing the procedures, and outcomes for data synthesis. Furthermore, future studies should also aim to develop a core outcome set for these interventions which include adverse events to assess the safety of LSSS (e.g., hyponatremia and hyperkalaemia). The absence of data on adverse event occurrences suggests the need for additional research (in relation to the use of LSSS), especially given that older adults and those on specific classes of drugs (used to treat hypertension) are at a greater risk of negative outcomes. The use of validated measurement instruments would also assist future studies to define specific adverse events (such as hyperkalaemia) and would subsequently improve the identification of adverse events during trials. Thirdly, it is unclear whether using LSSS consistently lowers sodium intake or whether it causes dietary adjustment through behavioural changes. Further research is required to determine the mechanism of how LSSS increases potassium intake (when potassium containing LSSS are used) and decreases sodium consumption over time. Finally, due to a dearth of literature, further research (high quality) is required to examine the benefits and any harms associated with the use of LSSS (replacing ordinary salt) in pregnant women and children (Brand et al. 2022).

### CPD reflective questions

- What populations may be at an increased risk of adverse events when considering replacing salt with low-sodium salt supplementation?
- What are the strengths and weaknesses of the systematic review?
- How does the evidence differ between children and adults when assessing the effectives of low-sodium salt supplementation compared to regular salt?

## Figures and Tables

**Table 1:** LSSS intervention compared to regular salt in adults (≥ 18 years) in the general population

Outcome	Relative effect / mean difference (95% CI)	No. of studies	Grade (certainty of evidence)	Comments
Blood pressure control	RR 2.12, 95% CI 1.32 to 3.41	2 RCTs	Very low	The evidence suggests that blood pressure control improved in adults receiving LSSS when compared to those receiving regular salt
Cardiovascular events: non-fatal stroke	RR of 0.90, 95% CI 0.80 to 1.01	3 RCTs	Moderate	On average, LSSS may slightly reduce non-fatal stroke events in adults when compared to regular salt
Cardiovascular events: non-fatal acute coronary syndrome	RR 0.70, 95% CI 0.52 to 0.94	1 RCT	Moderate	On average, LSSS slightly reduced non-fatal acute coronary syndrome in adults when compared to regular salt
Cardiovascular mortality	RR 0.77, 95% CI 0.60 to 1.00	3 RCTs	Moderate	On average, LSSS slightly reduced cardiovascular mortality events in adults when compared to regular salt
All-cause mortality	RR 0.89, 95% CI 0.83 to 0.95	5 RCTs	NR	On average, there was slightly reduced all-cause mortality events in adults receiving LSSS compared to those receiving regular salt
Antihypertensive medication use	RR 0.80, 95% CI 0.67 to 0.95	4 RCTs	NR	Antihypertensive medication use was lower in adults receiving the LSSS compared to those receiving regular salt
Microalbuminuria	RR 0.67, 95% CI 0.53 to 0.84	2 RCTs	NR	Microalbuminuria occurred less in adults receiving LSSS compared to those receiving regular salt
Macroalbuminuria	RR 0.48, 95%CI 0.16 to 1.39	1 RCT	NR	There was no evidence of difference in macroalbuminuria within adults who received LSSS compared with those receiving regular salt
Stroke mortality	RR 0.64, 95% CI 0.33 to 1.25	2 RCTs	Very low	There was no evidence of difference in stroke mortality within adults who received LSSS compared with those receiving regular salt
Hyperkalaemia	RR 1.04, 95% CI 0.46 to 2.38	5 RCTs	Moderate	There was no evidence of difference in hyperkalaemia within adults who received LSSS, compared with those receiving regular salt
Hypertension	RR 0.97, 95% CI 0.90 to 1.03	1 RCT	Low	There was no evidence of difference in hypertension for adults receiving LSSS compared to regular salt
Cardiovascular events: various	RR 1.22, 95% CI 0.49 to 3.04	5 RCTs	Very low	There was no evidence of difference in cardiovascular events within groups who received LSSS compared with those receiving regular salt
Hypokalaemia	NR	1 RCT	Very low	There was no evidence of difference in hypokalaemia within groups who received LSSS in adults, compared with those receiving regular salt
Change in blood potassium	MD 0.12, 95% CI 0.07 to 0.18	6 RCTs	Moderate	On average, LSSS slightly increased blood potassium in adults compared to regular salt
Change in systolic blood pressure (SBP, mmHg)	MD -4.76 mmHg, 95% CI -6.01 to - 3.50	20 RCTs	Moderate	On average, LSSS slightly reduced SBP in adults compared to regular salt
Change in diastolic blood pressure (DBP, mmHg)	MD -2.43 mmHg, 95% CI -3.50 to - 1.36	19 RCTs	Moderate	On average, LSSS slightly reduced DBP in adults, compared to regular salt
Change in 24-h urinary potassium excretion	MD 11.44 mmol (450 mg) potassium/24-h, 95% CI 7.62 to 15.26 mmol/24-h	11 RCTs	NR	On average, there was a change in 24-h urinary potassium excretion favouring adults receiving LSSS compared to those receiving regular salt
Change in total blood cholesterol	MD -0.31 mmol/L, 95% CI -0.74 to 0.12	4 RCTs	NR	There was no evidence of difference in change in total blood cholesterol for adults receiving LSSS compared to regular salt
Change in blood triglycerides	MD -0.11 mmol/L, 95% CI -0.91 to 0.69	5 RCTs	NR	There was no evidence of difference in change in blood triglycerides for adults receiving LSSS compared to regular salt

* LSSS- Low sodium salt substitutes, MD- mean difference, RR- Risk Ratio, NR- Not reported, CI- Confidence Interval, RCT- Randomised control trial, Very Low- The true effect is probably markedly different from the estimated effect, Low- The true effect might be markedly different from the estimated effect, Moderate- the authors believe that the true effect is probably close to the estimated effect.

**Table 2:** LSSS intervention compared to regular salt in children (2 to < 18 years) in the general population

Outcome	Mean difference (95% CI)	No. of studies	Grade (certainty of evidence)	Comments
Change in diastolic blood pressure (DBP, mmHg)	MD 1.28 mmHg, 95% CI -1.56 to 4.12	1 RCT	Very low	There was no evidence of difference in changes in DBP within groups who received bread containing LSSS compared to those receiving bread containing regular salt
Change in systolic blood pressure (SBP, mmHg)	MD 0.12 mmHg, 95% CI -4.41 to 4.64	1 RCT	Very Low	There was no evidence of difference in changes in SBP within groups who received bread containing LSSS compared to those receiving bread containing regular salt
Growth changes (e.g., BMI-for-age)	MD 0.94 kg/m^2^, 95% CI 0.85 to 1.03	1 RCT	NR	There was slightly greater reduction in BMI within groups who received bread containing LSSS compared to those receiving bread containing regular salt
Change in 24-h urinary sodium excretion	MD 14.60 mmol (336mg) sodium/24-h, 95%CI -11.22 to 40.42 mmol/24- h	1 RCT	NR	There was no evidence of difference in changes of 24-h urinary sodium excretion in the group who received bread containing LSSS compared to those receiving bread containing regular salt
Change in 24-h urinary potassium excretion	MD 4.10 mmol (160 mg) potassium/24-h, 95% CI - 5.13 to 13.33 mmol/24-h	1 RCT	NR	There was no evidence of difference in changes of 24-h urinary potassium excretion in the group who received bread containing LSSS compared to those receiving bread containing regular salt

* LSSS= Low sodium salt substitutes, MD= mean difference, RR= Risk Ratio, NR= Not reported, CI-Confidence Interval, RCT= Randomised control trial. Very Low- The true effect is probably markedly different from the estimated effect.

**Table 3:** Critical appraisal of the review by Brand et al, 2022

AMSTAR 2 items	Responses
^1.^ Did the research questions and inclusion criteria for the review include the components of PICO?	Yes – The study outlined the PICO’s within the methods section
^2.^ Did the report of the review contain an explicit statement that the review methods were established prior to the conduct of the review and did the report justify any significant deviations from the protocol?	Yes – The protocol was registered on the Cochrane Database of Systematic Reviews.
^3.^ Did the review authors explain their selection of the study designs for inclusion in the review?	Yes – The authors provided a detailed rationale on page 10.
^4.^ Did the review authors use a comprehensive literature search strategy?	Yes - Electronic searches of five databases, including MEDLINE, EMBASE and CINAHL were conducted.
^5.^ Did the review authors perform the study selection in duplicate?	Yes – Studies selection was independently conducted independently by two reviewers using Covidence.
^6.^ Did the review authors perform data extraction in duplicate?	Yes - Data extraction was conducted independently by two reviewers.
^7.^ Did the review authors provide a list of excluded studies and justify the exclusions?	Yes - The author provided reasons for exclusion and listed the studies in an appendix on page 180.
^8.^ Did the review authors describe the included studies in adequate details?	Yes – Two table were used to detail the characteristics of included studies.
^9.^ Did the review authors use a satisfactory technique for assessing the risk of bias in the individual studies that were included in the review?	Yes - two reviewers independently assessed the methodological quality of the included studies, and the GRADE approach was also used.
^10.^ Did the review authors report on the sources of funding for the studies included in the review?	Yes – The authors reported funding sources for each study on page 19 to 20.
^11.^ If meta-analysis was performed did the review authors use appropriate methods for statistical combination of results?	Yes - Meta-analysis was conducted with appropriate methods using random effects models.
^12.^ If meta-analysis was performed did the review authors assess the potential impact of RoB in individual studies on the results of the meta-analysis or other evidence synthesis?	Yes - The study conducted a sensitivity analysis to assess the potential impact of bias in individual trials on the results of the meta-analysis.
^13.^ Did the review authors account for RoB in individual studies when interpreting/discussing the results of the review?	Yes – the authors outline the risk of bias and GRADE assessment within each outcomes.
^14.^ Did the review authors provide a satisfactory explanation for and discussion of, any heterogeneity observed in the results of the review?	Yes – The authors included heterogeneity statistics within each meta-analysis.
^15.^ If they performed quantitative synthesis did the review authors carry out an adequate investigation of publication bias(small study bias) and discuss its likely impact on the results of the review?	Yes – A funnel plots in figure 4 and 5 suggested that publication bias could not be ruled out.
^16.^ Did the review authors report any potential sources of conflict of interest, including any funding they received for conducting the review?	Yes - The authors declared no competing interests

## Key Points

- Due to limitations of studies reporting adverse events and strict inclusion criteria’s, there is still uncertainty with regard to the safety profile of LSSS interventions. Further research is required to examine the benefits and harms associated with the use of LSSS (replacing ordinary salt) in at risk populations, pregnant women and children.
- Non-fatal stroke, non-fatal acute coronary syndrome, cardiovascular mortality, systolic blood pressure and diastolic blood pressure were significantly lower in adults who consumed LSSS compared to adults who consumed regular salt.
- There is no evidence of difference in instances of stroke mortality, hyperkalaemia, hypokalaemia, hypertension, or cardiovascular events (various) in adults who consumed LSSS compared to regular salt.
- There is no evidence of difference in blood pressure (DBP and SBP) within children who consumed LSSS compared to regular salt.
